# A new method based on YOLOv5 and multiscale data augmentation for visual inspection in substation

**DOI:** 10.1038/s41598-024-60126-2

**Published:** 2024-04-23

**Authors:** Junjie Chen, Siqi Pan, Yanping Chan, Yuedong Ni, Donghua Ye

**Affiliations:** State Grid Zhangzhou Power Supply Company, Zhangzhou, 363000 Fujian China

**Keywords:** Defect detection, Substation, YOLOv5, Multiscale data augmentation, Model pruning, Electrical and electronic engineering, Computer science, Information technology

## Abstract

Artificial intelligence has demonstrated notable advancements in the realm of visual inspection and defect detection in substations. Nevertheless, practical application presents challenges, with issues arising from the dynamic shooting environment and limited dataset resulting in suboptimal defect identification accuracy and instability. To address these concerns, a pioneering approach based on hybrid pruning YOLOv5 and multiscale data augmentation is proposed for enhancing defect detection in substations. Initially, an enhanced multiscale data augmentation method is proposed. The improved multiscale data augmentation mitigates the impact of the time-varying shooting environment on recognition accuracy and enhances defect detection precision. Subsequently, YOLOv5 is employed for training and detecting defects within multi-scale image data. To alleviate the potential destabilizing effects of YOLOv5’s large-scale parameters on model stability, a new model pruning method is implemented. This method strategically prunes parameters to bolster the model’s defect identification accuracy. The efficacy of the proposed methodology is evaluated through testing on substation defect images, confirming its effectiveness in enhancing defect detection capabilities.

## Introduction

Electric power is the key to ensure the normal operation of life, industry and other fields. Substation is the key node for completing power distribution in the power grid system. Given the complex and dynamic working environment, coupled with the intricate nature of substation equipment and the prevalence of various defects, the traditional manual inspection method encounters challenges such as long detection cycles and low detection accuracy. So traditional substation cannot meet the needs of the rapid development of the power industry^1,2^. Therefore, the intelligent substation brings new opportunities and challenges to the patrol inspection mode.

In recent years, several advanced monitoring devices have been developed for substation patrol inspection, such as unmanned aerial vehicle (UAV)^3^, infrared device^4^, robot^5^ and so on. Remote analysis and defect recognition of data images need the help of image recognition technology in the field of artificial intelligence^6,7^. The rapid development of this technology provides assistance to improve the automation of power systems. Some researchers have introduced image recognition technology into the power field. Ceron et al.^8^ used the Canny operator and direction adjustable filter to detect transmission lines. Khalayli et al.^9^ extracted the edge features of insulators with gray matrix, classified the features using a classifier, and detected the surface hydrophobicity of insulators. Jabid et al.^10^ proposed a method for estimating the rotation angle of all insulators and realized rotation invariance detection. In the aspect of substation equipment defect detection, the transformer overheating fault is detected by infrared thermal imager, and the equipment image features are extracted using the scale-invariant feature transform (SIFT) method, or the defect classification model is established. However, the above-mentioned methods need more advanced image processing technology to realize the extraction of defect features, and the applicability of the method is limited.

With the development of deep learning, image recognition technology has experienced a more rapid breakthrough. Deep learning can extract information with good performance from a large number of image data and automatically realize feature extraction^11–13^. Common deep learning methods include auto encoder^14^, restricted Boltzmann machine^15^, convolutional neural network^16^, and so on. Due to the unique advantages of convolutional neural network, such as weight sharing and sparse interaction, the target detection method based on convolutional neural network is becoming increasingly popular^17^. Sermanet et al.^18^ unified the classification and positioning tasks into a convolutional neural network through the OverFeat method, and used the multi-scale sliding window to detect each pixel position. The improvement enhanced the detection effect and secured the champion in the Imagenet large scale visual recognition challenge (ILSVRC) competition in 2013. Szegedy et al.^19^ proposed the concept of the deep convolution neural network framework, and built a Googlenet model based on it. This model can effectively improve the utilization rate of network parameters and won the champion of the ILSVRC competition in 2014. In 2015, He et al.^20^ proposed residual network (ResNet) to simplify the model unit, and solved the model degradation caused by network deepening. The model depth reached a depth of more than 100 layers, and the error rate was reduced to 3.57%. Additionally, Hinton et al.^21^ proposed a method to prevent model overfitting for small data sets, and the Kaiming He team proposed a multi-scale detection method of spatial pyramid pooling^22^.

In recent years, object detection algorithms based on deep learning have developed rapidly. Numerous neural network models with impressive performance have emerged. Based on the difference of network basic structure, these algorithms can be divided into two-stage detection network and one-stage detection network. The representative algorithm of the former is mainly R-CNN series^23,24^, and Yolo is the earliest and most representative one-stage network^25,26^. Dong et al.^27^ proposed an improved lightweight YOLOv5 method for vehicle detection. They utilized C3Ghost and Ghost modules to reduce the floating-point operations (FLOPs) during the feature confusion stage. The experimental results verified the effectiveness of the proposed method. Tan et al.^28^ utilized hollow convolution to resample the feature image. The ultralightweight subspace attention mechanism is used to improve the effect of the useful multiscale features. The proposed method is demonstrated on UAV images, resulting in a 5% improvement in accuracy. Kaushal^29^ proposed the rapid-YOLO that is an extension of YOLOv3 architecture. By adding extra detection layers and convolutional layers into YOLOv3, the proposed method reached high detection accuracy for detection of shadows. A non-maximum suppression technique using chaotic whale optimization is proposed to address false bounding box detections. The model was verified on rich experiments, and the results proved that the performance of the improved method is superior.

Although the deep learning method has made a lot of research results in the field of target detection, there is not much research in the field of substation defect detection. Therefore, it is prospective to study the application of deep learning in substation defect detection. In the actual scene of detecting defects in substations, there are mainly two problems: (1) the environment photographed will change with the operation of shooting tools, and the change of this environment will affect the quality of pictures, and then affect the accuracy of defect identification; (2) Since the field equipment does not often have defects, the amount of data of the defect fault images that can be captured is small, which leads to the model not being able to fully complete the training, thus reducing the accuracy of defect identification. In view of the above problems, a novel method combining pruning YOLOv5 and multi-scale data enhancement (HPYMDA) is proposed to complete the task of substation equipment defect identification. Firstly, a multiscale data augmentation method is introduced, employing distinct distributed initialization parameters for the design of convolution kernels. This approach facilitates the extraction of diverse multiscale features from the data, enhancing the richness of the input data. Consequently, this augmentation strategy contributes to an improved accuracy in defect identification. Then, the defect images of substation equipment collected by YOLOv5 training are used to complete the updating of model parameters, and the model is pruned by model pruning method to obtain a simplified model. Finally, the test set is input into the pruning model to test the effectiveness of the proposed method.

The main contributions in this paper are summarized as follows.

1. A novel method based on pruning YOLOv5 and multiscale data augmentation is proposed for substation patrol inspection. Considering real sense, the proposed method has strong adaptive power and high applicable value.
2. A multiscale data augmentation method is proposed for increasing data richness. A variety of different distribution methods are used to generate convolution kernel initialization weight parameters, and a variety of convolution kernels are generated by combining convolution kernels with different scales. The diversity of features is improved by extracting features through diversified convolution kernels.
3. A novel pruning method is proposed, the pruning of the YOLOv5 is determined according to the weight distribution of the feature map, and the channels at the edge of the weight distribution are removed, so as to retain the channels with obvious feature extraction effect. The weight is used to measure the function of the channel, which can cut the channel more accurately and improve the efficiency of the model pruning.

The rest of this paper is arranged as follows. The related works about YOLOv5 applied in object detection in Section “Related work”. In Section “Proposed method”, the details of the proposed method are described. The experimental results are discussed and analyzed in Section “Results and Discussions”. And in Section “Conculsion”, the conclusions are drawn from experimental results.

## Related work

### 1Object detection

Object detection is one of the typical application in the field of computer vision, widely utilized in face recognition, security monitoring, vehicle detection and so on. Target detection has evolved into two stages: traditional target detection and modern target detection combined with deep learning. Traditional target detection methods can be divided into artificial feature-based detection methods and motion based target detection methods^22^. The detection method based on artificial features mainly uses the representative features of the target itself to represent the appearance in the image, so as to complete the detection. These features include low-level features such as color and texture, and high-level features such as symmetry and shadow. The detection process is mainly divided into three steps: 1) select the area by using the sliding window; 2) Some feature extraction methods are adopted, such as scale transformation, histogram representation and Gaussian mixture model; 3) The classifier is trained and then the region in step 1 is identified. The feature extraction in the second step is very important, which is related to the accuracy of target detection. The feature selection is limited by the experience of the algorithm designer, and it is also very difficult in complex situations. Therefore, the feature-based detection algorithm lacks certain robustness and accuracy. At the same time, the information of texture, color and other images will be ignored by the shape feature expression, which also reduces the reliability of the application. Object detection algorithms based on deep learning have developed rapidly in recent years, and many network models with good performance have emerged.

According to the difference of network basic structure, it can be divided into two-stage detection network (two-stage) and one-stage detection network (one stage). The representative algorithm of the former is mainly R-CNN series, and YOLO is the earliest and most representative one-stage network. The two main model branches are to transform the target information through the network, extract abstract semantic information, and then complete target detection. However, the two-stage detection network represented by R-CNN has the following disadvantages: a large computational load and difficulty in achieving real-time performance. On the other hand, YOLO boasts strong real-time performance and high accuracy, and is a relatively good application model for target detection.

### YOLOv5

**Figure 1:**
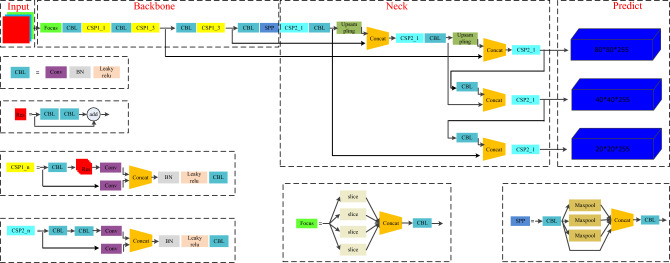
The structure of the YOLOv5s.

YOLO is a single-stage target detection method, which uses a single CNN model to complete the end-to-end target detection task. The updated version of YOLOv5^30^ is adopted in this paper. According to the depth of the network and the width of the feature map, it can be divided into four models, namely, YOLOv5s, YOLOv5m, YOLOv5l and YOLOv5x. The performance of these models is researched and obtained on the MS COCO test dataset as listed in Table 1. It can be seen that YOLOv5s model has smaller depth and feature map width, so it has faster computing speed. In contrast, YOLOv5x has higher accuracy. Due to the small amount of substation defect data, this paper uses YOLOv5s as the benchmark model. The structure of the YOLOv5s is shown in Fig. 1.

**Table 1:** The comparison performance of different models of YOLOv5.

Model	Size (pixels)	mAP (0.5)	mAP (0.5:0.95)	Params (Mb)	FLOPs (G)
YOLOv5s	640 × 640	55.2	36.4	7.2	16.7
YOLOv5m	640 × 640	63.3	45.5	21.2	50.3
YOLOv5l	640 × 640	65.9	47.2	46.3	114.4
YOLOv5x	640 × 640	68.3	51.3	87.5	217.4

YOLOv5 consists of the Input part, Backbone part, Neck network and Predict output part. Among them, the input end realizes an mosaic data enhancement, adaptive anchor box calculation and picture scaling. The adaptive anchor box calculation method compares the output prediction box with the real box based on the initial anchor box, calculates the gap and performs reverse update, and obtains the best anchor box value by continuously iteratively updating the parameters. The Backbone network is composed of a focus structure, convolution structure, C3 structure, spatial pyramid pooling structure and other modules. The focus module divides the input data into four parts, and each part is equivalent to two down sampling. After the four parts of data are cut along the channel dimension, a binary down sampling feature map is obtained through convolution operation, which realizes the function of increasing the channel dimension, reducing floating point operations (FLOPs) and improving the speed. The convolution structure includes the basic convolution unit, which is composed of convolution layer, batch normalization layer and activation function. The neck network includes FPN and pan structures, and plays the role of generating pyramid features. By generating feature maps of different scales, the feature representation ability is improved and the target detection effect is enhanced. In the prediction part, GIOU is used as the loss function of the boundary box on the output side to accelerate the regression prediction speed and obtain excellent results.

## Proposed method

### Improved multiscale data augmentation

Multiscale data augmentation is a classical data expansion method that utilizes the commonality of the same type data. In general, multiscale data augmentation methods are used to extract more information hidden in the raw data. According to the integrity of the raw data, the multiscale features can be divided into local features and global features. Obviously, the global features are extracted from a whole visual angle of the data. And the local features are explored via the local visual field. With the help of CNN, the different sizes of the convolutional kernels can extract different scale features. In supervised learning, a loss function is an accelerator for the convergence of the convolutional kernel. In the proposed method, however, the multiscale data augmentation method is only utilized as the data generator. Therefore, due to the randomness of convolutional kernel initialization, only a single size of convolutional kernels is used in data generation. A novel diversified multiscale data augmentation is proposed to improve the richness of the generation data.

**Figure 2:**
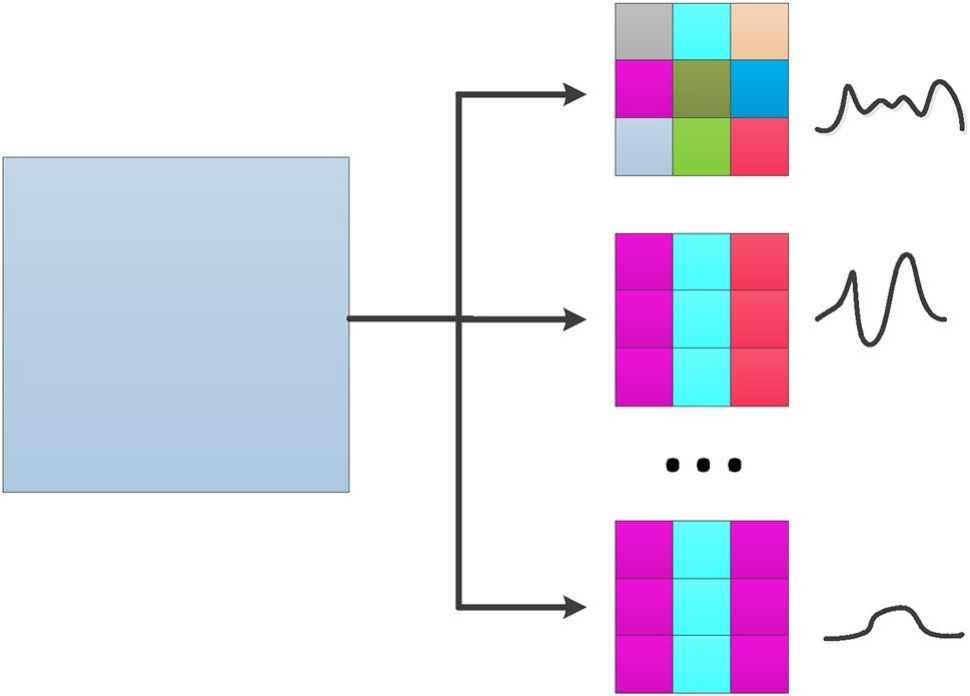
The different initialization rules of the improved multiscale data augmentation method.

Four initialization rules are designed for convolutional kernel, including random initialization, gaussian initialization, wavelet transform and the plus rule. The details are shown in Fig. 2.

The random initialization is to set the parameters of convolutional kernel in range of {0,1}.

Gaussian initialization uses gaussian kernel function to generate initialization parameters with the length of convolutional kernel. The formula is defined as follows:

1 $$Gaussian\left(x\right)=\frac{1}{\sqrt{2\pi }\sigma }{\text{exp}}\left(-\frac{{(x-\mu )}^{2}}{2{\sigma }^{2}}\right)$$

where $\mu$ is the mean of random variable x, and $\sigma$ is the standard deviation of x.

The wavelet transform is adopted to initialize the parameters of the convolutional kernel. The initialization formula is defined as follows:

2 $$WT\left(a,\tau \right)=\frac{1}{\sqrt{a}}{\int }_{-\infty }^{\infty }f\left(t\right)*\varphi \left(\frac{t-\tau }{a}\right)dt$$

where a is the scale and $\tau$ is the translation.

The plus rule applies plus operation to confuse above three initialization rules, and it is defined as follows:

3 $$plus=random+Gaussian+WT$$

### Pruning YOLOv5

The big parameter storage and strong computing capabilities are indispensable for the implementation of the YOLOv5. However, edge devices in an industrial application are usually unable to meet the demand. In addition, data scale of the industrial scene is smaller than the life scene, and the current mainstream neural network cannot perform well with small data. Therefore, it is necessary to compress the model capacity, and reduce the size of the model without excessive loss of identification accuracy. Common pruning ideas are mainly divided into uniform channel pruning, convolution channel pruning and automatic pruning. The latter two kinds of pruning are designed for complex network structure and difficult detection tasks. Therefore, in this paper, the uniform channel pruning is adopted. The aim of the uniform channel pruning is to trim the convolutional kernel of the fixed channel at a proportion. The proportion is usually set by experience without considering the importance of different feature maps. Therefore, in this paper, a new uniform channel pruning method based on weight distribution of feature maps is proposed.

A normal distribution is considered to constrain the proportion. It is defined as follows:

4 $$Normal\left(x\right)=\frac{1}{\sqrt{2\pi }\sigma }{\text{exp}}\left(-\frac{{(x-\mu )}^{2}}{2{\sigma }^{2}}\right)$$

### Measurement indicators

In common, to estimate the performance of the different object detection methods, precision, recall, mAP0.5, mAP0.5:0.95, average detection processing time, model size, FLOPs and parameter amount are utilized as the indicators. According to the desired result of the proposed method, precision, recall, mAP0.5 and mAP0.5:0.95 are selected as the main indicators.

Precision is calculated as the proportion of the number of the positive samples correctly predicted to the number of correctly predicted as positive samples. It is defined as follows:

5 $$Precision=\frac{TP}{TP+FP}$$

Recall is calculated as the proportion of the number of the positive samples correctly predicted to the sum number of the positive samples.

6 $$Recall=\frac{TP}{TP+FN}$$

where TP denotes the number of positive samples correctly identified, FP denotes the number of negative examples incorrectly classified, and FN is the number of positive samples that are incorrectly classified.

7 $$AP={\int }_{0}^{1}P\left(R\right)d R$$

8 $$mAP=\frac{{\sum }_{i=1}^{N}{AP}_{i}}{N}$$

where mAP0.5 refers to the average AP of all categories when intersection of union (IoU) is set to 0.5, and mAP0.5:0.95 refers to the average mAP at different IoU thresholds. The IoU ranges from 0.5 to 0.95 with a step size of 0.05.

Given the prediction box D, its coverage area is defined as area (D). The truth box is G, and its coverage area is area (G). Then the IoU is defined as follows:

9 $$IoU= \frac{area(D)\cap area(G)}{area(D)\cup area(G)}$$

where the overlap and union ratio IoU represents the degree of overlap between the prediction frame and the truth frame, and is a measure of the quality of the generated prediction frame.

## Results and discussions

### Experimental setting

**Figure 3:**
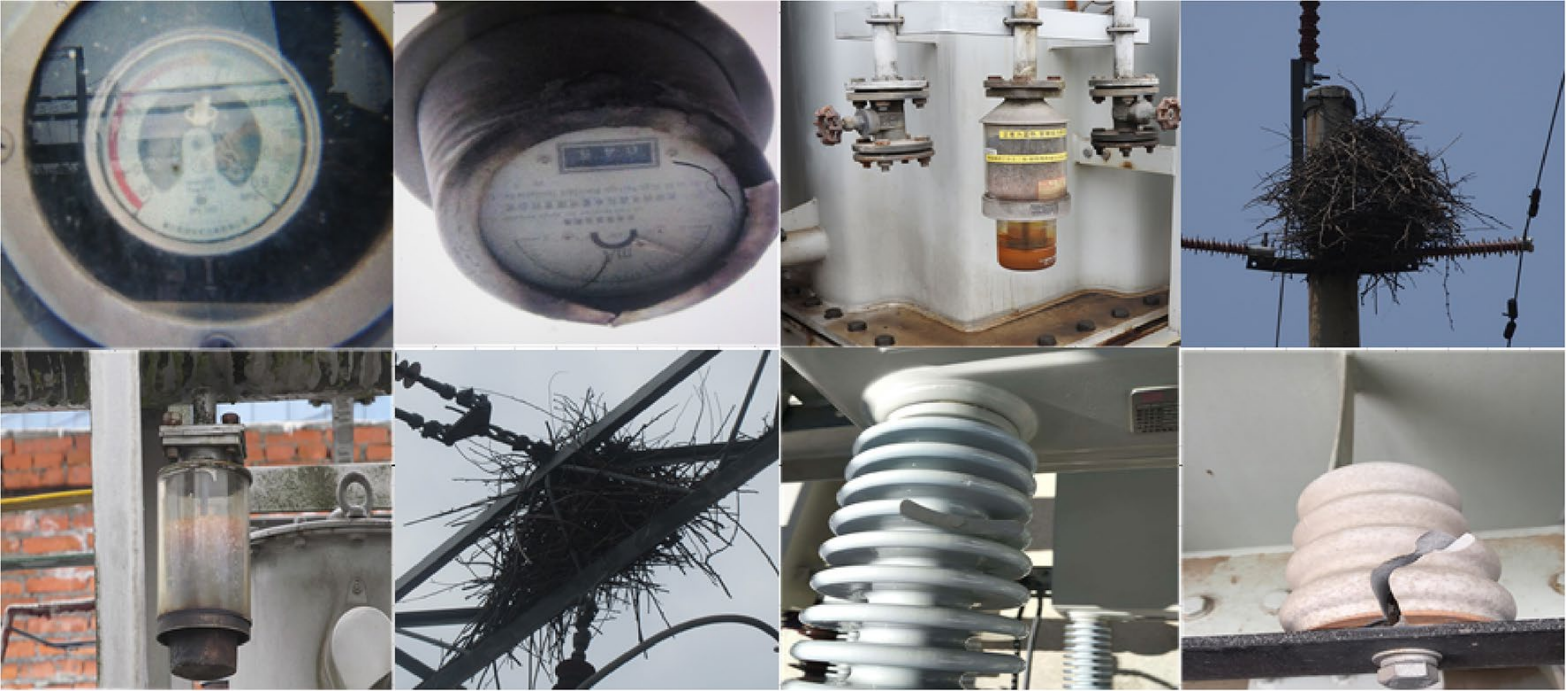
The dataset obtained by UAV from substation.

**Figure 4:**
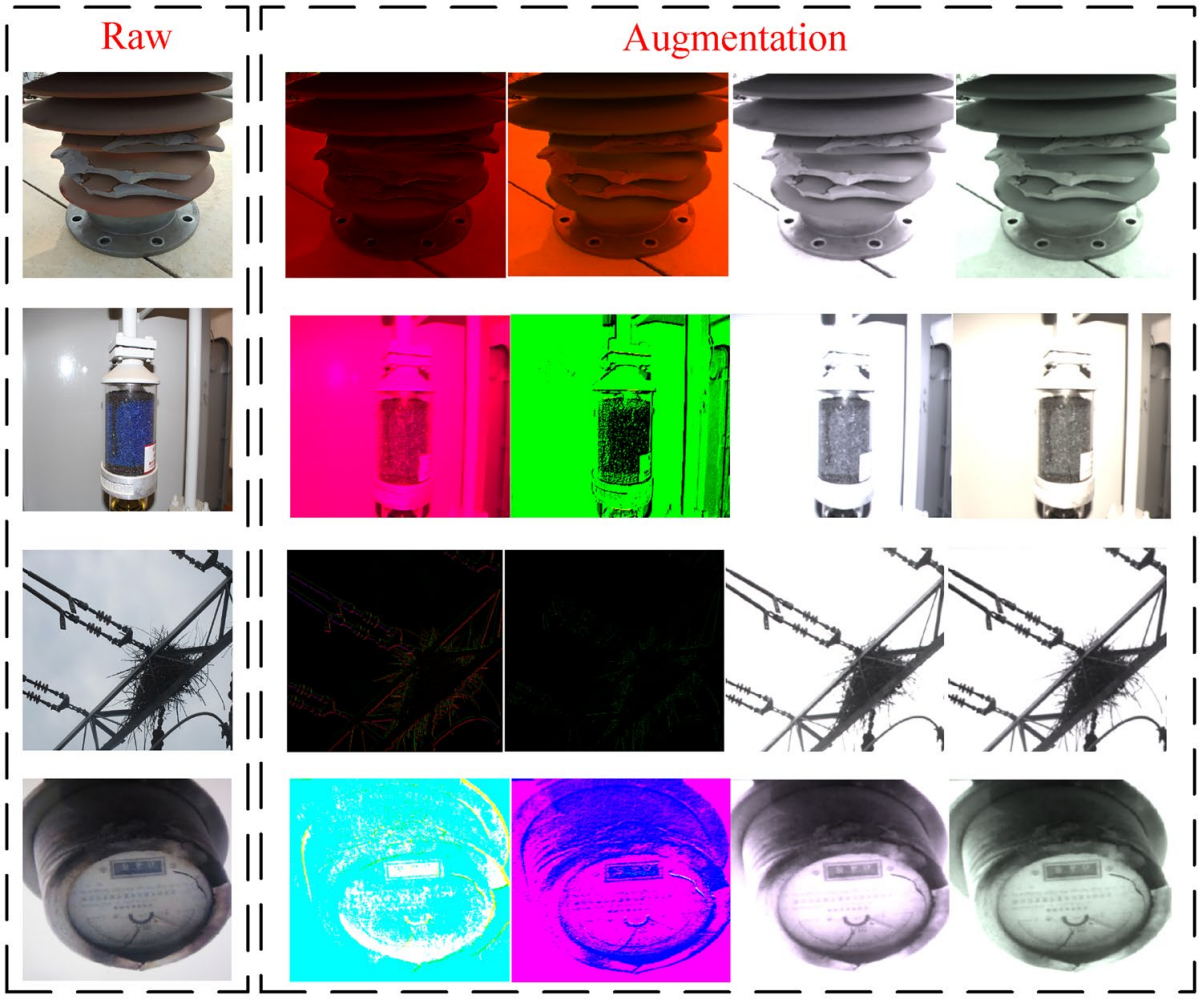
The dataset processed by improved multiscale data augmentation.

The experimental data includes the data collected from the substation and the data crawled by the network. The dataset includes five substation defects: fuzzy dial (f_d), damaged silica gel (dm_sg), insulator rupture (i_r), bird nest (b_n) and discolored silica gel (d_sg). The acquired image size is not the standard 640 * 640 * 3, so it needs to be trimmed to shape the image to meet the size of the model input. The constructed substation defect detection dataset contains five categories of defects with a total of 7440 images. To train the model for substation defect detection, the dataset is divided into a training set, a validation set, and a testing set at a proportion of 8:1:1. Then, 5952 images are randomly selected as the training set, and the validation set and testing set are consisting of 744 images respectively. The dataset is shown in Fig. 3. Before inputting into the pruning YOLOv5s model, the data set is first expanded by the proposed multi-scale enhancement method, and the effect of some pictures after enhancement is shown in Fig. 4.

In addition, YOLOv5s will use Mosaic method to enhance the data of the obtained images, and conduct diversified processing on the input data by changing brightness, contrast and rotation, so as to enrich the data set and improve the training accuracy of the model.

The training process of the proposed model is run under the Windows operating system and the PyTorch framework. The software environment is Python3.8.12. The CPU used to train the datasets is Intel(R) Xeon(R) E5-2630V3 CPU @2.4 GHz 32G.

During the training phase, the Adam algorithm is used to update the weight of the model. And the learning rate is set to 0.001, the batch size is set to 16, the epochs is set to 200.

### Result analysis

**Figure 5:**
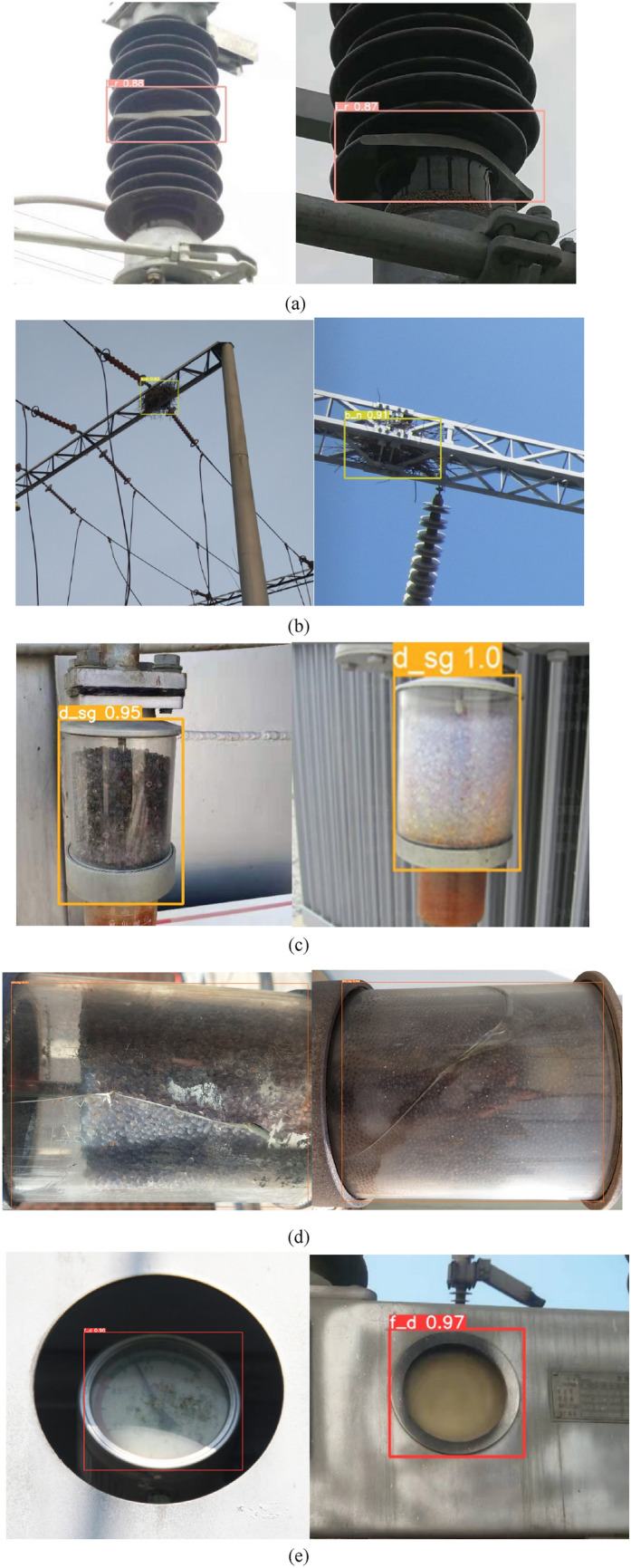
The detection result of different defects. (**a**) insulator rupture (**b**) bird nest (**c**) discolored silica gel (**d**) damaged silica gel (**e**) fuzzy dial.

**Figure 6:**
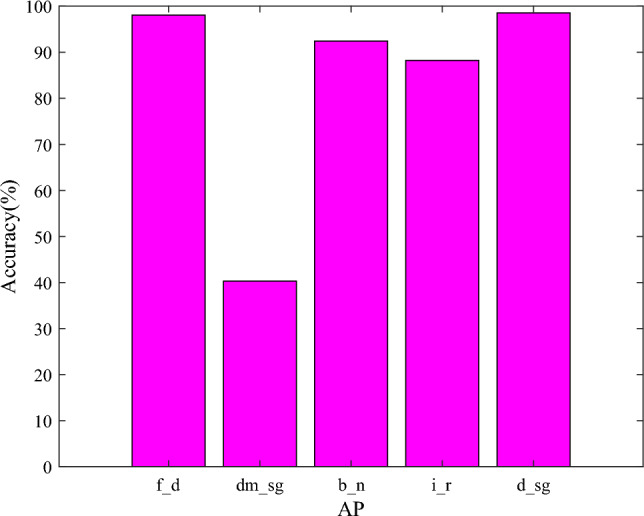
The single-type AP of different defects.

The detection results of different defects are shown in Fig. 5. Compared to Fig. 5a–e, we can see that the detection result of the damaged silica gel is the worst of the five defects. To show the result visually, the single-type AP of five defects obtained from the proposed method is listed in Fig. 6. From the result, we can see that the single-type AP of the discolored silica gel is the highest of the five defects with 98.54% and the detection result of the damaged silica gel is the worst with 40.32%. The main reason is that the damaged silica gel presents different texture structures, which are usually confused by obvious color. In addition, the damaged silica gel is often made of transparent glass, so it is difficult to distinguish crackles from other objects. As for the discolored silica gels, they are the same in the shape. The changing colors of silica gels are distinguishable.

Similarly, the single-type AP of the fuzzy dial is higher than the damaged dial. Because the fuzzy dial is only the color change in nature, but the damaged dial is changing in the structure. The changing structure increases identification difficulty and causes unsatisfactory results.

### Comparison experiment

In order to verify the effectiveness and improved effect of the proposed method, comparison experiments are implemented with six methods: YOLOv5s, pruning YOLOv5s, multiscale data augmentation + YOLOv5s, MSA-YOLOv5^31^, Faster R–CNN^32^ and the proposed method.

The implementation details of other methods are shown as follows.

1. YOLOv5s: In this method, images are input into the YOLOv5s without preprocessing stage. The learning rate is set to 0.001.
2. Pruning YOLOv5s: The proposed model pruning method is used to modify the YOLOv5s. The learning rate is set to 0.001.
3. Multiscale data augmentation + YOLOv5s: Compared with the proposed method, this method lacks pruning and improved multiscale data augmentation modules. The learning rate is set to 0.001.

The structure of MSA-YOLOv5 and Faster R–CNN are the same as originally proposed by the literature.

The comparison results are shown in Table 2. The results directly indicates that (1) The proposed pruning method can reduce the number of parameters and accelerate convergence of model with only a little loss at the accuracy. (2) The improved multiscale data augmentation shows nice performance, and the results proof the strong feature learning capacity of the improved multiscale data augmentation.

**Table 2:** The comparison measurement indictors of different methods.

Method	mAP (0.5)	mAP (0.5:0.95)	Params (Mb)	FLOPs (G)
YOLOv5s	72.2	53.7	7.2	24.3
Pruning YOLOv5s	71.4	52.5	6.4	22.5
Multiscale data augmentation + YOLOv5s	81.2	60.4	7.4	23.3
MSA-YOLOv5	80.2	58.3	13.39	20.4
Faster R–CNN	77.3	54.4	41.12	17.7
HPYMDA (ours)	83.5	62.3	6.7	22.4

Comparing YOLOv5s and pruning YOLOv5s, the proposed model pruning method reduces model size with a difference of 0.8Mb. Moreover, the mAP(0.5) only reduces by 0.7%. Therefore, the pruning method is effective for model pruning. With the improved multiscale data augmentation method, the mAP(0.5) increases by about 10%. The improved technique is more useful than the common multiscale data augmentation method. The powerful abilities of feature learning of the improved multiscale data augmentation are verified on the substation defect detection.

## Conculsion

In this paper, a new method named HPYMDA based on pruning YOLOv5 and multiscale data augmentation is proposed for substation defect detection. A novel multiscale data augmentation is proposed. Different data distribution rules are applied for generating diversified weights as the initialization of the convolutional kernel. Combined with different initializations with different scale kernel sizes, the feature maps extracted via convolutional kernel are so abundant that improving the identification accuracy of the proposed method. A model pruning method based on the weight proportion of different feature maps is proposed for cutting parameters of the YOLOv5. Compared with simple and rude model pruning methods by reducing the number of channels, the proposed pruning method implements parameters reduction reasonably. The effectiveness of the proposed method is verified on the dataset collected from a substation, and the results indicate that the HPYMDA is useful and valuable for substation defect detection in real application.

### Supplementary Information

Supplementary Information.

## Data Availability

The datasets generated during and/or analysed during the current study are available from the corresponding author on reasonable request.
