# Supplementary material for: A new method based on YOLOv5 and multiscale data augmentation for visual inspection in substation
Source: Sci Rep. 2024 Apr 23;14:9362. doi: 10.1038/s41598-024-60126-2 (PMC11039448; doi:10.1038/s41598-024-60126-2)
Supplement: Supplementary file 1 — Supplementary Information. [file 41598_2024_60126_MOESM1_ESM.pdf]

Due to large capacity (2GB), the dataset consisting of *images* was compressed and uploaded to the netdisk:

Link：https://pan.baidu.com/s/12zyhQrGA9xBR4FT7QlJzug

Extraction code：6666
